# Supplementary material for: Could prokinetic agents protect long-term nasogastric tube-dependent patients from being hospitalized for pneumonia? A nationwide population-based case-crossover study
Source: PLoS One. 2021 Apr 5;16(4):e0249645. doi: 10.1371/journal.pone.0249645 (PMC8021154; doi:10.1371/journal.pone.0249645)
Supplement: S1 Table — (DOCX) [file pone.0249645.s001.docx]

S1 Table. The association between prokinetics exposure and pneumonia admission (time period changed to 14 days)

|  |  | Crude OR | 95% Cl | | | *P* value | Adjusted OR^a^ | 95% Cl | | | *P* value |
| --- | --- | --- | --- | --- | --- | --- | --- | --- | --- | --- | --- |
| General population | All prokinetics | 1.31 | (0.92 |  | 1.86) | 0.1318 | 1.31 | (0.91 |  | 1.87) | 0.1468 |
| n=639 | Metoclopramide | 1.15 | (0.69 |  | 1.90) | 0.6017 | 1.09 | (0.65 |  | 1.82) | 0.7489 |
|  | Mosapride | 1.21 | (0.58 |  | 2.53) | 0.6119 | 1.28 | (0.60 |  | 2.75) | 0.5280 |
|  | Domperidone | 1.34 | (0.75 |  | 2.38) | 0.3187 | 1.37 | (0.76 |  | 2.45) | 0.2960 |
|  |  |  |  |  |  |  |  |  |  |  |  |
| Age ≧ 65 years old | All prokinetics | 1.37 | (0.94 |  | 1.99) | 0.1047 | 1.33 | (0.90 |  | 1.96) | 0.1521 |
| n=564 | Metoclopramide | 1.05 | (0.61 |  | 1.83) | 0.8509 | 0.97 | (0.56 |  | 1.69) | 0.9258 |
|  | Mosapride | 1.24 | (0.57 |  | 2.68) | 0.5935 | 1.27 | (0.57 |  | 2.86) | 0.5605 |
|  | Domperidone | 1.44 | (0.78 |  | 2.65) | 0.2431 | 1.44 | (0.77 |  | 2.69) | 0.2505 |
|  |  |  |  |  |  |  |  |  |  |  |  |
| Male | All prokinetics | 1.12 | (0.67 |  | 1.88) | 0.6587 | 1.11 | (0.65 |  | 1.91) | 0.7013 |
| n=291 | Metoclopramide | 1.11 | (0.51 |  | 2.39) | 0.7922 | 1.04 | (0.47 |  | 2.28) | 0.9314 |
|  | Mosapride | 1.40 | (0.46 |  | 4.30) | 0.5578 | 1.79 | (0.54 |  | 5.97) | 0.3428 |
|  | Domperidone | 0.73 | (0.31 |  | 1.75) | 0.4810 | 0.71 | (0.29 |  | 1.70) | 0.4385 |
|  |  |  |  |  |  |  |  |  |  |  |  |
| Female | All prokinetics | 1.49 | (0.93 |  | 2.41) | 0.0996 | 1.53 | (0.94 |  | 2.51) | 0.0891 |
| n=348 | Metoclopramide | 1.17 | (0.60 |  | 2.30) | 0.6422 | 1.18 | (0.59 |  | 2.33) | 0.6431 |
|  | Mosapride | 1.09 | (0.41 |  | 2.88) | 0.8669 | 1.07 | (0.40 |  | 2.91) | 0.8897 |
|  | Domperidone | 2.38 | (1.05 |  | 5.42) | 0.0388* | 2.69 | (1.14 |  | 6.35) | 0.0238* |
|  |  |  |  |  |  |  |  |  |  |  |  |
| Diabetes Mellitus | All prokinetics | 0.81 | (0.52 |  | 1.28) | 0.3690 | 0.77 | (0.48 |  | 1.22) | 0.2668 |
| n=391 | Metoclopramide | 0.68 | (0.35 |  | 1.35) | 0.2716 | 0.62 | (0.31 |  | 1.23) | 0.1693 |
|  | Mosapride | 0.61 | (0.22 |  | 1.67) | 0.3342 | 0.57 | (0.20 |  | 1.58) | 0.2788 |
|  | Domperidone | 1.00 | (0.50 |  | 2.02) | 1.0000 | 1.06 | (0.52 |  | 2.18) | 0.8731 |
|  |  |  |  |  |  |  |  |  |  |  |  |
| Stroke | All prokinetics | 1.22 | (0.84 |  | 1.79) | 0.2962 | 1.22 | (0.82 |  | 1.80) | 0.3314 |
| n=510 | Metoclopramide | 1.24 | (0.71 |  | 2.14) | 0.4503 | 1.18 | (0.68 |  | 2.05) | 0.5618 |
|  | Mosapride | 1.23 | (0.57 |  | 2.62) | 0.5999 | 1.26 | (0.57 |  | 2.78) | 0.5730 |
|  | Domperidone | 0.93 | (0.48 |  | 1.79) | 0.8265 | 0.95 | (0.49 |  | 1.84) | 0.8678 |
|  |  |  |  |  |  |  |  |  |  |  |  |
| Parkinsonism | All prokinetics | 1.83 | (0.95 |  | 3.53) | 0.0733 | 1.86 | (0.91 |  | 3.76) | 0.0869 |
| n=166 | Metoclopramide | 1.59 | (0.62 |  | 4.06) | 0.3337 | 1.51 | (0.56 |  | 4.08) | 0.4131 |
|  | Mosapride | 1.44 | (0.34 |  | 6.11) | 0.6186 | 1.97 | (0.42 |  | 9.20) | 0.3887 |
|  | Domperidone | 2.31 | (0.71 |  | 7.47) | 0.1634 | 2.19 | (0.64 |  | 7.49) | 0.2124 |

**P* value < 0.05.

^a^ Odds ratios adjusted for antipsychotic agents, benzodiazepine-receptor agonists, histamine H2-blockers, proton pump inhibitors, statins, angiotensin receptor blockers, and angiotensin-converting enzyme inhibitors exposure
