# Supplementary material for: Could prokinetic agents protect long-term nasogastric tube-dependent patients from being hospitalized for pneumonia? A nationwide population-based case-crossover study
Source: PLoS One. 2021 Apr 5;16(4):e0249645. doi: 10.1371/journal.pone.0249645 (PMC8021154; doi:10.1371/journal.pone.0249645)
Supplement: S2 Table — (DOCX) [file pone.0249645.s002.docx]

S2 Table. The association between prokinetics exposure and pneumonia admission (time period changed to 45 days)

|  |  | Crude OR | 95% Cl | | | *P* value | Adjusted OR^a^ | 95% Cl | | | *P* value |
| --- | --- | --- | --- | --- | --- | --- | --- | --- | --- | --- | --- |
| General population | All prokinetics | 1.27 | (0.93 |  | 1.72) | 0.1302 | 1.29 | (0.94 |  | 1.76) | 0.1093 |
| n=639 | Metoclopramide | 0.88 | (0.59 |  | 1.30) | 0.5117 | 0.91 | (0.61 |  | 1.35) | 0.6223 |
|  | Mosapride | 1.47 | (0.82 |  | 2.62) | 0.1956 | 1.35 | (0.74 |  | 2.46) | 0.3267 |
|  | Domperidone | 1.51 | (0.95 |  | 2.39) | 0.0817 | 1.57 | (0.98 |  | 2.52) | 0.0599 |
|  |  |  |  |  |  |  |  |  |  |  |  |
| Age ≧ 65 years old | All prokinetics | 1.33 | (0.96 |  | 1.85) | 0.0839 | 1.36 | (0.98 |  | 1.90) | 0.0695 |
| n=564 | Metoclopramide | 0.93 | (0.61 |  | 1.41) | 0.7239 | 0.96 | (0.62 |  | 1.47) | 0.8381 |
|  | Mosapride | 1.49 | (0.82 |  | 2.69) | 0.1867 | 1.34 | (0.73 |  | 2.47) | 0.3487 |
|  | Domperidone | 1.64 | (1.01 |  | 2.67) | 0.0457* | 1.72 | (1.05 |  | 2.83) | 0.0325* |
|  |  |  |  |  |  |  |  |  |  |  |  |
| Male | All prokinetics | 1.16 | (0.75 |  | 1.81) | 0.4978 | 1.18 | (0.76 |  | 1.85) | 0.4594 |
| n=291 | Metoclopramide | 1.00 | (0.57 |  | 1.74) | 1.0000 | 1.06 | (0.60 |  | 1.87) | 0.8472 |
|  | Mosapride | 0.89 | (0.40 |  | 2.00) | 0.7856 | 0.80 | (0.34 |  | 1.87) | 0.6042 |
|  | Domperidone | 0.83 | (0.40 |  | 1.73) | 0.6228 | 0.82 | (0.40 |  | 1.72) | 0.6048 |
|  |  |  |  |  |  |  |  |  |  |  |  |
| Female | All prokinetics | 1.37 | (0.90 |  | 2.09) | 0.1480 | 1.43 | (0.92 |  | 2.22) | 0.1144 |
| n=348 | Metoclopramide | 0.78 | (0.45 |  | 1.34) | 0.3622 | 0.79 | (0.45 |  | 1.39) | 0.4137 |
|  | Mosapride | 2.65 | (1.09 |  | 6.44) | 0.0320* | 2.41 | (0.97 |  | 6.02) | 0.0595 |
|  | Domperidone | 2.29 | (1.24 |  | 4.23) | 0.0081* | 2.55 | (1.35 |  | 4.82) | 0.0040* |
|  |  |  |  |  |  |  |  |  |  |  |  |
| Diabetes Mellitus | All prokinetics | 0.95 | (0.63 |  | 1.42) | 0.7841 | 0.95 | (0.63 |  | 1.44) | 0.8071 |
| n=391 | Metoclopramide | 0.62 | (0.37 |  | 1.03) | 0.0667 | 0.64 | (0.38 |  | 1.08) | 0.0967 |
|  | Mosapride | 1.10 | (0.52 |  | 2.32) | 0.7989 | 0.93 | (0.43 |  | 2.02) | 0.8607 |
|  | Domperidone | 1.10 | (0.61 |  | 1.98) | 0.7605 | 1.18 | (0.64 |  | 2.16) | 0.6000 |
|  |  |  |  |  |  |  |  |  |  |  |  |
| Stroke | All prokinetics | 1.31 | (0.93 |  | 1.84) | 0.1276 | 1.36 | (0.96 |  | 1.94) | 0.0850 |
| n=510 | Metoclopramide | 0.94 | (0.61 |  | 1.44) | 0.7727 | 1.01 | (0.65 |  | 1.58) | 0.9552 |
|  | Mosapride | 1.58 | (0.84 |  | 2.99) | 0.1587 | 1.42 | (0.73 |  | 2.75) | 0.2966 |
|  | Domperidone | 1.27 | (0.75 |  | 2.15) | 0.3662 | 1.34 | (0.78 |  | 2.29) | 0.2838 |
|  |  |  |  |  |  |  |  |  |  |  |  |
| Parkinsonism | All prokinetics | 2.62 | (1.43 |  | 4.82) | 0.0019* | 2.43 | (1.31 |  | 4.49) | 0.0047* |
| n=166 | Metoclopramide | 1.32 | (0.64 |  | 2.71) | 0.4544 | 1.37 | (0.66 |  | 2.87) | 0.4016 |
|  | Mosapride | 1.66 | (0.56 |  | 4.93) | 0.3648 | 1.63 | (0.49 |  | 5.42) | 0.4230 |
|  | Domperidone | 3.10 | (1.29 |  | 7.45) | 0.0113* | 3.18 | (1.26 |  | 8.00) | 0.0142* |

**P* value < 0.05.

^a^ Odds ratios adjusted for antipsychotic agents, benzodiazepine-receptor agonists, histamine H2-blockers, proton pump inhibitors, statins, angiotensin receptor blockers, and angiotensin-converting enzyme inhibitors exposure
