# Supplementary material for: Could prokinetic agents protect long-term nasogastric tube-dependent patients from being hospitalized for pneumonia? A nationwide population-based case-crossover study
Source: PLoS One. 2021 Apr 5;16(4):e0249645. doi: 10.1371/journal.pone.0249645 (PMC8021154; doi:10.1371/journal.pone.0249645)
Supplement: S3 Table — (DOCX) [file pone.0249645.s003.docx]

S3 Table. The association between prokinetics exposure and pneumonia admission (washout period changed to 7 days)

|  |  | Crude OR | 95% Cl | | | *P* value | Adjusted OR^a^ | 95% Cl | | | *P* value |
| --- | --- | --- | --- | --- | --- | --- | --- | --- | --- | --- | --- |
| General population | All prokinetics | 1.06 | (0.78 |  | 1.45) | 0.7098 | 1.10 | (0.80 |  | 1.51) | 0.5758 |
| n=639 | Metoclopramide | 0.93 | (0.62 |  | 1.40) | 0.7304 | 0.93 | (0.61 |  | 1.41) | 0.7177 |
|  | Mosapride | 1.18 | (0.64 |  | 2.20) | 0.5944 | 1.20 | (0.64 |  | 2.25) | 0.5712 |
|  | Domperidone | 1.17 | (0.71 |  | 1.92) | 0.5486 | 1.28 | (0.77 |  | 2.12) | 0.3472 |
|  |  |  |  |  |  |  |  |  |  |  |  |
| Age ≧ 65 years old | All prokinetics | 1.10 | (0.79 |  | 1.54) | 0.5702 | 1.14 | (0.81 |  | 1.61) | 0.4400 |
| n=564 | Metoclopramide | 0.94 | (0.60 |  | 1.45) | 0.7681 | 0.93 | (0.59 |  | 1.46) | 0.7607 |
|  | Mosapride | 1.29 | (0.68 |  | 2.43) | 0.4404 | 1.29 | (0.67 |  | 2.45) | 0.4468 |
|  | Domperidone | 1.19 | (0.70 |  | 2.00) | 0.5266 | 1.29 | (0.76 |  | 2.21) | 0.3442 |
|  |  |  |  |  |  |  |  |  |  |  |  |
| Male | All prokinetics | 0.82 | (0.52 |  | 1.30) | 0.3966 | 0.84 | (0.52 |  | 1.37) | 0.4870 |
| n=291 | Metoclopramide | 0.74 | (0.40 |  | 1.35) | 0.3187 | 0.69 | (0.36 |  | 1.31) | 0.2570 |
|  | Mosapride | 0.76 | (0.31 |  | 1.87) | 0.5563 | 0.80 | (0.33 |  | 1.99) | 0.6372 |
|  | Domperidone | 0.73 | (0.31 |  | 1.75) | 0.4810 | 0.88 | (0.37 |  | 2.12) | 0.7829 |
|  |  |  |  |  |  |  |  |  |  |  |  |
| Female | All prokinetics | 1.33 | (0.87 |  | 2.04) | 0.1903 | 1.34 | (0.86 |  | 2.09) | 0.1902 |
| n=348 | Metoclopramide | 1.15 | (0.66 |  | 2.01) | 0.6306 | 1.13 | (0.64 |  | 2.02) | 0.6717 |
|  | Mosapride | 1.88 | (0.76 |  | 4.62) | 0.1703 | 1.86 | (0.75 |  | 4.64) | 0.1809 |
|  | Domperidone | 1.51 | (0.81 |  | 2.81) | 0.1981 | 1.52 | (0.80 |  | 2.88) | 0.1969 |
|  |  |  |  |  |  |  |  |  |  |  |  |
| Diabetes Mellitus | All prokinetics | 0.78 | (0.52 |  | 1.18) | 0.2438 | 0.80 | (0.52 |  | 1.22) | 0.2950 |
| n=391 | Metoclopramide | 0.65 | (0.38 |  | 1.13) | 0.1290 | 0.65 | (0.37 |  | 1.14) | 0.1330 |
|  | Mosapride | 0.83 | (0.36 |  | 1.95) | 0.6717 | 0.80 | (0.34 |  | 1.90) | 0.6185 |
|  | Domperidone | 0.87 | (0.46 |  | 1.64) | 0.6700 | 0.93 | (0.49 |  | 1.77) | 0.8281 |
|  |  |  |  |  |  |  |  |  |  |  |  |
| Stroke | All prokinetics | 0.94 | (0.66 |  | 1.33) | 0.7246 | 0.98 | (0.68 |  | 1.40) | 0.8924 |
| n=510 | Metoclopramide | 0.92 | (0.58 |  | 1.44) | 0.7014 | 0.93 | (0.58 |  | 1.49) | 0.7668 |
|  | Mosapride | 1.17 | (0.60 |  | 2.31) | 0.6422 | 1.20 | (0.60 |  | 2.39) | 0.6058 |
|  | Domperidone | 0.82 | (0.46 |  | 1.46) | 0.5011 | 0.89 | (0.50 |  | 1.61) | 0.7102 |
|  |  |  |  |  |  |  |  |  |  |  |  |
| Parkinsonism | All prokinetics | 2.43 | (1.27 |  | 4.64) | 0.0074* | 2.46 | (1.26 |  | 4.82) | 0.0084* |
| n=166 | Metoclopramide | 2.16 | (0.91 |  | 5.17) | 0.0825 | 2.08 | (0.84 |  | 5.17) | 0.1129 |
|  | Mosapride | 1.41 | (0.45 |  | 4.45) | 0.5577 | 1.45 | (0.43 |  | 4.95) | 0.5526 |
|  | Domperidone | 2.05 | (0.79 |  | 5.32) | 0.1408 | 2.10 | (0.80 |  | 5.50) | 0.1318 |

**P* value < 0.05.

^a^ Odds ratios adjusted for antipsychotic agents, benzodiazepine-receptor agonists, histamine H2-blockers, proton pump inhibitors, statins, angiotensin receptor blockers, and angiotensin-converting enzyme inhibitors exposure
