# Supplementary material for: Could prokinetic agents protect long-term nasogastric tube-dependent patients from being hospitalized for pneumonia? A nationwide population-based case-crossover study
Source: PLoS One. 2021 Apr 5;16(4):e0249645. doi: 10.1371/journal.pone.0249645 (PMC8021154; doi:10.1371/journal.pone.0249645)
Supplement: S4 Table — (DOCX) [file pone.0249645.s004.docx]

S4 Table. The association between prokinetics exposure and pneumonia admission (washout period changed to 15 days)

|  |  | Crude OR | 95% Cl | | | *P* value | Adjusted OR^a^ | 95% Cl | | | *P* value |
| --- | --- | --- | --- | --- | --- | --- | --- | --- | --- | --- | --- |
| General population | All prokinetics | 1.05 | (0.77 |  | 1.45) | 0.7452 | 1.09 | (0.78 |  | 1.51) | 0.6170 |
| n=639 | Metoclopramide | 0.85 | (0.57 |  | 1.29) | 0.4503 | 0.86 | (0.57 |  | 1.31) | 0.4932 |
|  | Mosapride | 1.58 | (0.84 |  | 2.99) | 0.1587 | 1.75 | (0.91 |  | 3.35) | 0.0940 |
|  | Domperidone | 1.19 | (0.72 |  | 1.98) | 0.4899 | 1.23 | (0.73 |  | 2.05) | 0.4374 |
|  |  |  |  |  |  |  |  |  |  |  |  |
| Age ≧ 65 years old | All prokinetics | 1.11 | (0.79 |  | 1.56) | 0.5576 | 1.15 | (0.80 |  | 1.63) | 0.4540 |
| n=564 | Metoclopramide | 0.88 | (0.56 |  | 1.36) | 0.5534 | 0.88 | (0.56 |  | 1.38) | 0.5833 |
|  | Mosapride | 1.72 | (0.88 |  | 3.35) | 0.1116 | 1.88 | (0.95 |  | 3.71) | 0.0682 |
|  | Domperidone | 1.25 | (0.73 |  | 2.15) | 0.4081 | 1.27 | (0.74 |  | 2.20) | 0.3906 |
|  |  |  |  |  |  |  |  |  |  |  |  |
| Male | All prokinetics | 0.74 | (0.47 |  | 1.17) | 0.1961 | 0.77 | (0.48 |  | 1.25) | 0.2870 |
| n=291 | Metoclopramide | 0.64 | (0.35 |  | 1.16) | 0.1398 | 0.64 | (0.34 |  | 1.18) | 0.1530 |
|  | Mosapride | 0.81 | (0.33 |  | 2.01) | 0.6515 | 0.96 | (0.38 |  | 2.44) | 0.9368 |
|  | Domperidone | 0.66 | (0.30 |  | 1.46) | 0.3043 | 0.67 | (0.30 |  | 1.52) | 0.3396 |
|  |  |  |  |  |  |  |  |  |  |  |  |
| Female | All prokinetics | 1.50 | (0.95 |  | 2.36) | 0.0788 | 1.51 | (0.94 |  | 2.42) | 0.0863 |
| n=348 | Metoclopramide | 1.11 | (0.64 |  | 1.94) | 0.7030 | 1.10 | (0.62 |  | 1.94) | 0.7523 |
|  | Mosapride | 3.63 | (1.27 |  | 10.37) | 0.0163* | 4.29 | (1.44 |  | 12.77) | 0.0089* |
|  | Domperidone | 1.90 | (0.96 |  | 3.78) | 0.0663 | 1.86 | (0.92 |  | 3.76) | 0.0831 |
|  |  |  |  |  |  |  |  |  |  |  |  |
| Diabetes Mellitus | All prokinetics | 0.75 | (0.49 |  | 1.14) | 0.1780 | 0.76 | (0.49 |  | 1.18) | 0.2167 |
| n=391 | Metoclopramide | 0.63 | (0.36 |  | 1.10) | 0.1025 | 0.64 | (0.36 |  | 1.14) | 0.1276 |
|  | Mosapride | 1.07 | (0.44 |  | 2.65) | 0.8767 | 1.09 | (0.44 |  | 2.70) | 0.8560 |
|  | Domperidone | 0.85 | (0.45 |  | 1.58) | 0.5985 | 0.88 | (0.46 |  | 1.66) | 0.6835 |
|  |  |  |  |  |  |  |  |  |  |  |  |
| Stroke | All prokinetics | 0.93 | (0.65 |  | 1.32) | 0.6766 | 0.95 | (0.66 |  | 1.37) | 0.7826 |
| n=510 | Metoclopramide | 0.90 | (0.57 |  | 1.41) | 0.6474 | 0.93 | (0.58 |  | 1.47) | 0.7475 |
|  | Mosapride | 1.58 | (0.79 |  | 3.14) | 0.1976 | 1.73 | (0.86 |  | 3.51) | 0.1265 |
|  | Domperidone | 0.78 | (0.44 |  | 1.38) | 0.3874 | 0.77 | (0.43 |  | 1.39) | 0.3854 |
|  |  |  |  |  |  |  |  |  |  |  |  |
| Parkinsonism | All prokinetics | 2.82 | (1.39 |  | 5.69) | 0.0039* | 3.05 | (1.46 |  | 6.35) | 0.0030* |
| n=166 | Metoclopramide | 1.83 | (0.80 |  | 4.19) | 0.1540 | 1.85 | (0.76 |  | 4.46) | 0.1735 |
|  | Mosapride | 1.79 | (0.55 |  | 5.82) | 0.3315 | 2.37 | (0.65 |  | 8.62) | 0.1890 |
|  | Domperidone | 2.73 | (0.99 |  | 7.56) | 0.0528 | 2.55 | (0.91 |  | 7.13) | 0.0753 |

**P* value < 0.05.

^a^ Odds ratios adjusted for antipsychotic agents, benzodiazepine-receptor agonists, histamine H2-blockers, proton pump inhibitors, statins, angiotensin receptor blockers and angiotensin-converting enzyme inhibitors exposure
